# Empathy and cooperation vary with gender in Chinese junior high school adolescents

**DOI:** 10.1002/pchj.705

**Published:** 2023-11-09

**Authors:** Qin Wu, Weiwei Bu, Dong Lin, Liying Cui, Aruna Wu, Hehui Zou, Chen Gu

**Affiliations:** ^1^ School of psychology Shanghai Normal University Shanghai China

**Keywords:** cooperative behavior, cooperative propensity, empathy, gender difference, junior high school adolescents

## Abstract

Previous research on the relationship between empathy and subcategories of prosocial behavior, specifically cooperation, has shown inconsistent findings. It has also paid limited attention to gender differences in the impact of empathy. Therefore, this study examined the relationship between empathy and cooperation in Chinese junior high school adolescents, and the gender differences, through three studies. In Study 1, 448 eighth‐grade adolescents (age = 12–15 years, 55.1% males) completed the Interpersonal Reactivity Index and Cooperative Propensity Rating Scale; the results showed that adolescent empathy was positively associated with cooperative propensity, and this association was significantly higher for males than for females. Study 2 used longitudinal data from 246 eighth‐grade adolescents (age = 12–15 years, 54.5% males) to further support the positive association between empathy and cooperation propensity and the gender differences found in Study 1. Study 3 employed the public goods dilemma to examine the effects of empathic states on the cooperative behavior of 157 eighth‐grade adolescents (age = 13–16 years, 48% males) by evoking empathy. Using different research methods, this study revealed a facilitative relationship between empathy and cooperation and demonstrated that empathy was more predictive of cooperation among male than among female adolescents.

## INTRODUCTION

In recent years, global public events, such as COVID‐19, ecological imbalances, climate change, and resource shortages, have become significant challenges for humankind. Cooperation is an approach by which humanity can collectively address global public issues, serving as the foundation for social, national, and global sustainable development (Lv, 2020). Furthermore, these global challenges foster a sense of shared destiny among humans, allowing us to cherish each other and empathize with people in similar circumstances, thereby facilitating a heightened capacity for empathy to some extent. Can empathy foster the willingness to cooperate with others and thus become an effective way to solve public problems?

Most researchers have defined empathy as the ability to understand and share the feelings of others and to respond appropriately to their situations (Liu et al., 2009; Preston & de Waal, 2002). Empathy in research is often categorized into empathic traits and states. Empathic traits refer to the personality context and can be represented by a multidimensional structure that includes fantasy, empathic attention, perspective taking, and personal distress (Davis, 1994). Empathic states refer to specific contexts and are often elicited in behavioral experiments through reading materials, watching videos, and recall writing. In contemporary society's complex interpersonal and social networks, a high level of empathy is beneficial for individuals regarding handling interpersonal relationships harmoniously and it increases the likelihood of understanding others' feelings and responding sensitively (Lv, 2020). Ultimately, possessing a high level of empathy could foster interpersonal cooperation.

Cooperation refers to relatively stable, persistent, and implicit personality dispositions (i.e., cooperative propensity) as well as to immediate, impulsive, and outward behavioral decisions (i.e., cooperative behavior; Liu, 2021). Cooperative propensity refers to an individual's tendency to decide whether or to what extent to cooperate in a potentially cooperative situation (Pang & Cheng, 2001). Cooperative behavior refers to the behavior or intention of individuals or groups to coordinate activities for common goals and promote the realization of certain results that are beneficial to themselves and others (Henrich & Henrich, 2006).

According to the empathy–altruism hypothesis, empathy has motivational and informational functions that allow individuals to more easily perceive others' needs and assess others' perspectives, and that empathic concern can stimulate an individuals' altruistic motivation to engage in prosocial behaviors (Batson, 2011; Batson et al., 1991). Moreover, research on the impact of empathy on prosocial behaviors has increased, and multiple studies have consistently validated the empathy–altruism hypothesis by demonstrating that empathy is an important cause of individuals' prosocial or altruistic behaviors (Carrizales et al., 2021; de Waal, 2008; Ding & Lu, 2016; Qu et al., 2022; Wang & Wu, 2020).

However, previous studies focused mostly on prosocial or altruistic behaviors, and relatively little attention has been paid to the direct relationship between empathy and interpersonal cooperation. While cooperative, helping, and altruistic behaviors fall under the prosocial umbrella, the motivations behind these behaviors differ (Lv, 2020; Wu & Cui, 2020). Acts of helping and altruism are often driven by individuals' benevolent and altruistic motives, which aim to assist others. Conversely, cooperative behavior is motivated by mutualism or win–win outcomes, where both parties engage in collaborative activities toward a common goal. In terms of behavioral outcomes, helping and altruistic acts benefit others, whereas cooperation maximizes the mutual benefits for oneself and others (Wu & Cui, 2020). Moreover, the relationship between the interacting parties in different prosocial behaviors varies and can be reflected through game rules and behavioral outcomes. In typical cooperation paradigms, such as the public goods dilemma, the interacting parties are clearly seen as interdependent, and individual behavioral outcomes depend on the actions of the other participants. In contrast, the dictator game, which is often used to measure altruistic behavior, is typically considered to involve independent relationships (Lv, 2020). Thus, different types of prosocial behaviors exhibit multifaceted differences. However, existing research on the relationship between empathy and prosocial behaviors has failed to differentiate between the types of prosocial behaviors, with a limited focus on the direct relationship between empathy and cooperation. This significantly limits the generalizability of the empathy–altruism hypothesis.

Furthermore, the conclusions of the few studies that explored the relationship between empathy and cooperation resulted in significant controversies. For instance, Batson and Moran (1999) manipulated empathy using reading materials and found that the empathic group had significantly higher levels of cooperation in the prisoner's dilemma than did the non‐empathic group. Rumble et al. (2010) used the dictator game to demonstrate that empathy motivated generous behavior and was an effective tool for maintaining or enhancing cooperation. Similarly, Xu et al. (2012) demonstrated that empathy promoted cooperation in the prisoner's dilemma and fostered forgiveness after a partner's defection. However, another experiment in the same study (Experiment 2 in Xu et al. 2012) found that empathy did not have a significant impact on cooperation. Furthermore, Lv (2020) conducted a meta‐analysis of 11 studies and found that empathy and cooperation exhibited a mixed and unstable correlation, rather than a significant positive relationship. In addition, Lv (2020) conducted four studies using college students as participants and consistently demonstrated that empathy did not effectively promote individual cooperative behavior. These findings challenge the previous research conclusions that supported the idea that empathy promotes cooperation. These inconsistent results highlight the importance of conducting a comprehensive examination to assess the stability of the relationship between empathy and cooperation.

Moreover, based on the previous studies, the following doubts about the relationship between empathy and cooperation remain.

The extant research on empathy and cooperation has predominantly focused on the relationship between empathic states and cooperative behavior, with limited investigation of trait empathy and few comprehensive examination of individual cooperation by integrating cooperative propensity and behavior. The integrative model of decision‐making in social dilemmas (Parks et al., 2013) suggests that individual differences in trait empathy influence the attachment system, allowing empathy to serve as a distal factor that both impacts individual trait cooperation and further influences decision‐making processes and behavioral choices in specific situations. Moreover, according to the dynamic model of empathy (Liu et al., 2009), empathy is a psychological phenomenon in interpersonal interactions and involves a dynamic and directional psychosocial process (Hoffman, 2002) that influences corresponding behavioral tendencies, motivations, and decisions. Therefore, both empathy and cooperation should be comprehensively examined at the individual trait and situational state levels.

Furthermore, studies have found that individual cooperative behavior is influenced by a combination of intrinsic factors, such as social values, emotions and personality, and extrinsic factors, such as group characteristics, task structures, and reward and punishment systems (Balliet et al., 2011; Zhou et al., 2020), this diversity enables individuals with high levels of cooperative propensity not to necessarily engage in cooperative behavior in their actual behavioral choices; instead, cooperative propensity is moderately correlated with cooperative behavior (Kohn, 1991). Thus, the combined exploration of empathic traits and states and cooperative tendencies and behaviors constitutes an important part of testing the stable relationship between empathy and cooperation.

In addition, the previous research on the relationship between empathy and interpersonal cooperation has focused mostly on college students (Batson & Moran, 1999; Lv, 2020; Rumble et al., 2010; Xu et al., 2012) or explored the effects of cooperation on children's empathy development (Brownell et al., 2002; Marcus et al., 1979). Adolescence is a sensitive period that involves increased complexity regarding social relationships and environments (Blakemore & Mills, 2014), and social interactions play a crucial role in adolescents' socialization processes. Empathy is a crucial aspect in social interactions, and individuals with lower empathetic ability may find it difficult to recognize and understand the emotions and feelings of others, which hinders their establishment of harmonious relationships with others (Nusantara et al., 2020). On the other hand, cooperation is a form of prosocial interaction and plays a vital role in individuals' integration into social groups (Cui et al., 2022). Hence, it is of paramount importance to focus on empathy and cooperation in adolescent groups.

Finally, previous studies have focused on gender differences in empathy and have provided empirical evidence and meta‐analytic findings indicating gender differences in empathy that are also influenced by age (Yan & Su, 2018). Specifically, studies have found no significant differences in empathy between males and females during the preschool stage, while the differences gradually widen from mid‐childhood to adolescence and peak during adolescence (Chen et al., 2014; Yan & Su, 2018). This is consistent with the gender role reinforcement theory, which suggests that gender differences increase between early and mid‐adolescence (Hill & Lynch, 1983; Van Der Graaff et al., 2018). These findings highlight the importance of exploring empathy‐related gender differences during adolescence. Moreover, the previous research has suggested that the interaction between empathy and prosocial behavior is stronger among females than among males (Van der Graaff et al., 2014, 2018), and that females have greater empathy than males (Chakrabarti & Baron‐Cohen, 2006). However, prior studies have also shown that males exhibit greater forgiveness and higher positive emotions and attitudes toward partner defection than females (Xu et al., 2012). Moreover, various components of empathy do not work in unison for males and females (Eisenberg et al., 2001). Therefore, there may be complex patterns of gender differences related to the impact of empathy on behavior during adolescence, and the specific manifestation of these differences requires further empirical investigation.

Therefore, this study comprehensively examined the trait and state aspects of empathy and cooperation among adolescents. It investigated the stability of the relationship between empathy and cooperation through three separate studies and explored the role of gender in this relationship. Specifically, Studies 1 and 2 adopted the empathic trait perspective. Study 1 used questionnaires to explore the relationship between empathy and cooperative propensity among Chinese junior high school adolescents, and preliminarily examined the role of gender in the relationship. From the statistical perspective, the cross‐sectional design obscured whether empathy was a predictor of cooperation. Study 2 investigated the predictive relationship between trait empathy and cooperative propensity, as well as the role of gender, using short‐term longitudinal data from the adolescent sample. Study 3 used the empathetic states perspective to further examine the effect of laboratory‐induced empathy on adolescents' cooperative behavior via the public goods dilemma and assessed the gender differences.

## STUDY 1

### Objective

The aim of Study 1 was to conduct a preliminary investigation into the relationship between adolescents' empathy and cooperative propensity from the empathic trait perspective, and the role of gender, through a questionnaire survey.

### Methods

#### Participants

Participants comprised Chinese adolescents in the eighth grade from three junior high schools (two in Shanghai and one in Suzhou). A total of 480 questionnaires were distributed, of which 448 (201 females [44.9%], 247 males [55.1%], *M*_age_ = 13.34 years, *SD*_age_ = 0.60, range: 12–15 years) were validated after eliminating those with missing and random answers, with an effective response rate of 93.3%. All data for this and the following studies were collected after approval from the Ethics Review Board of the Social Sciences Office of Shanghai Normal University. All participants provided written informed consent to participate.

#### Tools

The Chinese version of the Interpersonal Reactivity Index (IRI‐C), developed by Davis (1983) and revised by Zhang et al. (2010), was used to measure the level of trait empathy among the junior high school adolescents. The questionnaire consisted of 22 questions divided into four subscales: fantasy, empathic concern, perspective taking, and personal distress. A five‐point Likert scale was adopted, and the sum of the four subscales was the total score of empathy, with higher total scores indicating higher individual empathy levels. The IRI has been widely used to measure empathy in many psychological studies, and its factor structure has been confirmed for those aged 13 years and older (Hawk et al., 2013). In this study, Cronbach's *α* coefficient of the scale was .75.

The Adolescent Propensity to Cooperate Rating Scale was adapted from the cooperation subscale of the Cooperative and Competitive Personality Scale developed by Xie et al. (2006). The questionnaire consisted of 13 questions divided into three subscales: inclusiveness, reciprocity, and gregariousness. A five‐point Likert scale was adopted, with higher scores indicating higher individual cooperative propensity. In this study, Cronbach's *α* coefficient of the scale was .87, and were 0.64, 0.69, and 0.74 for the three subscales, respectively.

#### Data collection and processing

The questionnaires were distributed in the classes and collected on the spot. SPSS 25.0 and the PROCESS 3.0 plugin were used for the data entry, processing, and analysis.

### Results and analysis

#### Common method deviation test

This study performed Harman's single‐factor test to determine whether the results were affected by common method bias, which is generally considered to be a significant problem when a single factor accounts for more than 40% of the total variance among the measured variables (Harman, 1976). In this study, the most influential factor accounted for 19.70% of the total variance, suggesting that the common method bias effect was not a problem.

#### Results of descriptive statistics

Descriptive statistical analyses were conducted on empathy and cooperative propensity, as well as on the scores for the various cooperative propensity dimensions, among the eighth‐grade students of different genders. The results are shown in Table 1.

**TABLE 1:** Descriptive statistics and correlation analysis of each variable.

Variable	*M*	*SD*	1	2	3	4	5
1. Gender	0.55	0.50	‐				
2. Empathy	3.24	0.49	0.18***	‐			
3. Inclusiveness	4.02	0.68	0.05	0.31***	‐		
4. Reciprocity	3.14	0.79	−0.11*	0.22***	0.29***	‐	
5. Gregariousness	3.75	0.78	−0.09	0.19***	0.37***	0.62***	‐
6. Cooperative propensity	3.64	0.59	−0.07	0.30***	0.67***	0.83***	0.86***

*Note*: *N* = 448; gender (0 = male, 1 = female).

* *p* < .05.

*** *p* < .001.

The results indicate that empathy was positively correlated with cooperative propensity and its various dimensions among the junior high school adolescents, with correlation coefficients ranging from 0.19 to 0.31.

To further understand the association between empathy and cooperative propensity, and whether there were differences in the association between genders, this study conducted the analysis using Model 1 of the PROCESS macro for SPSS. It set gender as the moderator variable and used standardized bootstrapping (5000 samples) for empathy and cooperative propensity. The results are shown in Table 2.

**TABLE 2:** Association between empathy and cooperative propensity among junior high school adolescents, and the moderating role of gender.

Variable	Effect	*SE*	*t*	95% CI	
				LLCI	ULCI
Constant	0.06	0.07	2.48*	−0.07	0.19
Gender	−0.27	0.09	−3.16**	−0.44	−0.10
Empathy	0.40	0.06	6.39***	0.28	0.52
Empathy × Gender	−0.19	0.09	−2.18*	−0.35	−0.02

Abbreviations: CI, confidence interval; LLCI, lower limit of confidence interval; ULCI, upper limit of confidence interval.

* *p* < .05.

** *p* < .01.

**** *p* < .001.

**FIGURE 1:**
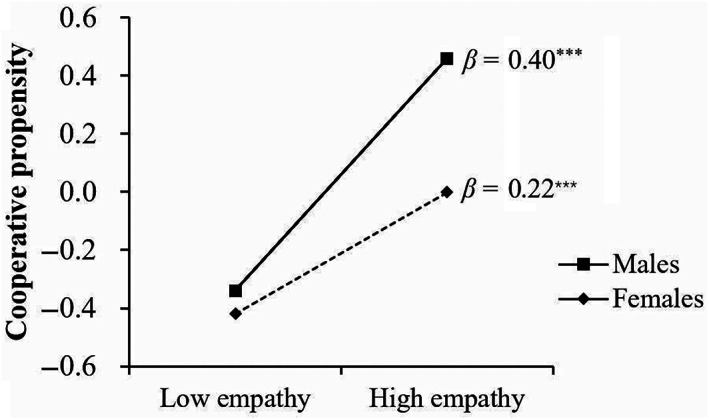
Gender differences in the association of empathy and cooperative propensity.

Empathy was significantly and positively associated with cooperative propensity (*β* = 0.40, *p* < .001). The effects of gender (*β* = −0.27, *p* = .002) and the two‐way interaction of empathy × gender (*β* = −0.19, *p* = .030) showed a significant association with cooperative propensity. This suggests that there were gender differences in the association of empathy and cooperative propensity. Specifically, the effect of empathy on cooperative propensity was significantly greater for males (*β* = 0.40, *t* = 6.395, *p* < .001, 95% confidence interval [CI] = [0.278, 0.524]) than for females (*β* = 0.22, *t* = 3.719, *p* < .001, 95% CI = [0.101, 0.329]). The moderating effects are shown in Figure 1.

## STUDY 2

### Objective

Study 1 examined the positive association between empathy and cooperative propensity among adolescents and found that the association was significantly higher for males than for females. However, this result was based on only a single questionnaire survey and did not consider the influence of other individual factors on the relationship. Therefore, to further optimize the investigation of the relationship between empathy and cooperative propensity and to conduct a focused survey on the eighth‐grade group that showed gender differences, Study 2 examined the longitudinal relationship between empathy and cooperative propensity using short‐term longitudinal data from eighth graders.

### Methods

#### Participants

Eighth graders from a junior high school in Suzhou were selected as participants; the interval between the two surveys was 4 months. A total of 246 valid questionnaires (121 females [49.2%], 125 males [50.8%], *M*_age_ = 13.42 years, *SD*_age_ = .66, range: 12–15 years) were collected at Time 1 (T1). Of the participants, 161 (65.4%) were local students, and 234 (95.1%) were from two‐parent families. Regarding their parents' education level, 2.4% of the participants' fathers had completed primary school education, 34.6% had received a junior high school education, 35.4% had received a senior high school education, 27.2% had obtained a college degree or higher, and 1 participant did not report their father's education level. Furthermore, 6.9% of the participants' mothers had completed primary school education, 35.8% had received a junior high school education, 37.8% had received a senior high school education, and 19.5% had obtained a college degree or higher. A total of 218 participants completed the Time 2 (T2) measurement (88.6%); 28 participants were missing at T2 owing to leave of absence, school transfer, sick leave, and other reasons. The missing and valid samples did not differ in terms of the father's and mother's education levels (*χ* ^2^(3) = 2.702, *p* = .440; *χ* ^2^(3) = 2.375, *p* = .498), family type (*χ* ^2^(1) = 0.116, *p* = .733), age (*t*(244) = 1.916, *p* = .056), T1 trait empathy (*t*(244) = −1.297, *p* = .196), and cooperative propensity (*t*(244) = −1.090, *p* = .277); however, males were more likely to be missing than females (*χ* ^2^(1) = 18.77, *p* < .001).

#### Tools

This study used the IRI‐C (the same as that used in Study 1; Cronbach's *α* coefficients were .79 and .84 for T1 and T2, respectively), and the Adolescent Propensity to Cooperate Rating Scale (the same as that used in Study 1; Cronbach's *α* coefficients were .79 and .81 for T1 and T2, respectively).

#### Data collection and processing

The questionnaires were distributed in the classes and collected on the spot. Descriptive statistical analysis of the variables was performed using SPSS 25.0. The gender differences in the impact of trait empathy on cooperation were examined using Model 1 in the PROCESS macro (Hayes, 2013).

### Results and analysis

#### Results of descriptive statistics

Pearson's correlation was used to analyze the correlation between empathy in T1 and cooperative propensity in T1 and T2. The results are shown in Table 3.

**TABLE 3:** Correlational results of T1 empathy and cooperation propensity at T1 and T2.

Variable	*M*	*SD*	1	2	3
1. T1 empathy	3.12	0.42	‐		
2. T1 cooperative propensity	3.67	0.51	0.27***	‐	
3. T2 cooperative propensity	3.46	0.62	0.29***	0.50***	‐

*Note*: N = 246.

*** *p* < .001.

The correlation coefficient between T1 and T2 cooperative propensity was 0.50 (*p* < .001). The correlation coefficient between T1 empathy and T1 cooperative propensity was 0.27 (*p* < .001), and the correlation coefficient between T1 empathy and T2 cooperative propensity was 0.29 (*p* < .001).

#### Gender differences in the effect of empathy on cooperative propensity

In the Model 1 analysis conducted via the PROCESS macro, T2 cooperative propensity was used as the dependent variable, T1 trait empathy was used as the independent variable, and gender was used as the moderator variable. Before data processing, all variables were standardized, and T1 cooperative propensity was included as a control variable. The analysis was performed using bias‐corrected percentile bootstrapping with 5000 resamples to calculate a 95% CI. The results showed that after controlling for gender and parents' education level, T1 cooperative propensity significantly predicted T2 cooperative propensity (*β* = 0.446, *SE* = 0.060, *p* < .001, 95% CI = 0.327 to 0.565), T1 empathy significantly predicted T2 cooperative propensity (*β* = 0.507, *SE* = 0.179, *p* = .005, 95% CI = 0.155 to 0.859), and the interaction effect of T1 empathy and gender on T2 cooperative propensity was significant (*β* = −0.249, *SE* = 0.119, *p* = .037, 95% CI = −0.482 to −0.015). These findings indicate that there were significant gender differences in the relationship between empathy and cooperative propensity.

**FIGURE 2:**
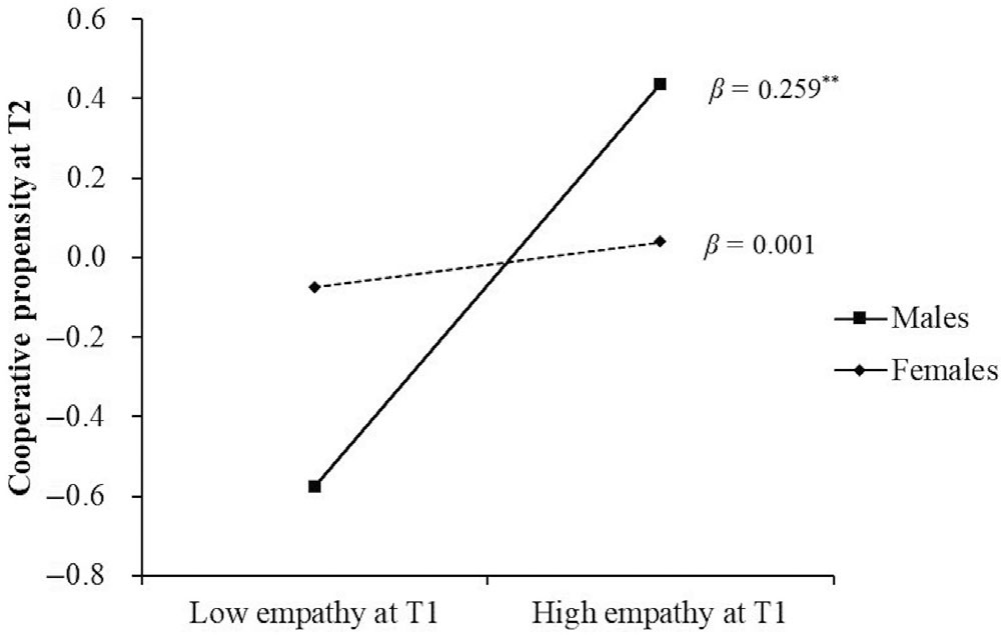
Gender differences in the predictive role of empathy on cooperative propensity.

In addition, we conducted a simple slope analysis controlling for participants' age and parents' education level to examine the relationship between empathy and cooperative propensity. The results show that T1 empathy significantly predicted T2 cooperative propensity for males (*β* = 0.259, *SE* = 0.078, *p* = .001, 95% CI = 0.105 to 0.413). However, the predictive effect of T1 empathy on T2 cooperative propensity was not significant for females (*β* = 0.001, *SE* = 0.092, *p* = .915, 95% CI = −0.171 to 0.191). The results are shown in Figure 2.

## STUDY 3

### Objective

Study 3 aimed to explore the effect of empathy on adolescents' cooperative behavior via the public goods dilemma from the empathic state perspective, and to seek further behavioral‐level evidence for the gender differences found in Studies 1 and 2.

In the setting of cooperation paradigms, the previous studies have primarily employed the prisoner's dilemma (Batson & Moran, 1999; Xu et al., 2012), the dictator game (Rumble et al., 2010), and the two‐person repeated public goods dilemma (Lv, 2020), and have often used virtual interaction partners (Lv, 2020; Rumble et al., 2010) or single‐round experimental designs (Batson & Moran, 1999; Xu et al., 2012). These paradigms are all based on dyadic, bidirectional interactions, and the interactive deception with virtual partners may influence the examination of empathy and cooperation dynamics (Lv, 2020). Moreover, multi‐round interactions simulate the interactive process of cooperative behavior in real‐life situations better than single‐round interactions. Therefore, this study adopted a multi‐round real interaction with four‐person groups to investigate cooperative behavior.

### Methods

#### Participants

Before conducting the experiment, this study adopted a moderate effect size (*f* = 0.25) to calculate the sample size using G*Power 3.1 (Faul et al., 2007). The sample size analysis of the analysis of variance (ANOVA; fixed effects, main effects, and interactions, *α* = 0.05, and 80% power) showed that a minimum of 128 participants were required.

Overall, 170 eighth‐grade adolescents at a junior high school in Suzhou participated in the study. According to the record of the experimental scene (mutual communication and negotiation of investment during the experiment), 13 invalid participants were excluded, leaving 157 participants (*M*_age_ = 14.19 years, *SD*_age_ = 0.63, range: 13–16 years), of which 81 (42 males [51.9%], 39 females [48.1%]) were placed in the experimental group and 74 (36 males [48.6%], 38 females [51.4%]) were placed in the control group.

#### Experimental design

This study used a 2 (gender: male, female) × 2 (group: experimental, control) between‐subjects design. The independent variable was manipulated by having participants from diverse groups read various materials to evoke empathy in the experimental group. The dependent variable was cooperative behavior, measured by the first‐round investment amount and the average of the six‐round investment amount in the public goods dilemma.

#### Experimental materials

##### State Empathic Response Scale

This study used the State Empathic Response Scale (Batson et al., 1991) to examine the effectiveness of the state empathy arousal in this study. The scale consisted of the following six adjectives expressing empathic emotional experiences: sympathetic, warm, compassionate, soft‐hearted, tender, and moved. The responses ranged from not at all (1) to extremely (7) and were averaged to form an index of self‐reported empathy (Cronbach's *α* = 0.95).

##### Reading materials for empathy arousal

To evoke state empathy, this study adopted the text‐reading method from the classical contextual elicitation paradigm (Graziano et al., 2007) used in previous studies (Lin et al., 2016). The participants in the experimental group read a 507‐word text about a freshman who had recently experienced a life change and needed help, which contained the evocation of personal pain and empathetic concern. The participants in the control group read a 501‐word text about three crucial factors that affected China's climate, which were selected from a paragraph in the grade 8 geography textbook.

##### Cooperative behavior

Referring to the step‐level public goods dilemma game task (Liu & Hao, 2015), this study used the Java programming language to create a web‐based version of the public goods investment game. After reading the game rules and completing three rule comprehension questions, the participants entered the game lobby and were randomly grouped with other players to form four‐person teams and engage in group investment. In each round, participants received 100 CNY (ChinaYuan) worth of game coins and deposited any amount they wished (CNY 0–100) into the public account created by the group. If the total amount invested in this round exceeded CNY 200, it was doubled and divided equally among each group member. If the total amount was less than CNY 200, the invested game coins were confiscated by the system. After each round of investment, the participants received feedback on their earnings in that round and the total amount in their personal account. The game included six rounds.

#### Experimental procedure

The experiment was conducted using the school's computer room and administered to 12–24 participants simultaneously (multiple groups could be formed). The participants were requested to read and sign the informed consent form and then complete a short pre‐test questionnaire. Then, all participants simultaneously read the corresponding textual materials on emotional elicitation and completed the state empathy questionnaire according to the instructional prompts. They then logged on to the public goods dilemma game page to complete the cooperative behavior measurement.

#### Data processing

This study used SPSS 25.0 for the data entry, processing, and analysis.

### Results and analysis

#### Effectiveness testing of the state empathy arousal

The results of the independent samples *t*‐test showed no significant difference in the level of trait empathy between the experimental and control groups (*t* = 0.95, *df* = 156, *p* = .358), excluding the interference of the initial trait empathy on the experimental manipulation.

Regarding the effectiveness evaluation of the emotion elicitation using the State Empathy Response Scale, the independent samples *t*‐tests showed that participants in the experimental group had significantly higher state empathy scores than those in the control group (*M*_experiment_ = 5.16 ± 1.60; *M*_control_ = 2.79 ± 1.58, *t* = 9.34, *p* < .001), suggesting that the study's experiments were effective for state empathy arousal.

#### Impact of state empathy on cooperative behavior in the public goods dilemma

Table 4 shows the descriptive statistics results of the first investment round and the average of the six rounds of investment in the public goods dilemma for the experimental and control groups.

**TABLE 4:** Experimental and control group participants' cooperative behavior in the public goods dilemma (*M* ± *SD*).

Cooperative behavior	Experimental group		Control group	
	Males (42)	Females (39)	Males (36)	Females (38)
First round of investments	61.00 ± 36.06	57.31 ± 30.34	40.31 ± 27.72	54.76 ± 30.53
Average of six rounds of investments	61.46 ± 27.79	58.25 ± 25.17	63.21 ± 28.05	63.77 ± 20.40

Controlling for cooperative propensity, trait empathy, and age, this study conducted a series of ANOVAs, with the group and gender as the independent variables and the first investment round and the average of the six investment rounds as the dependent variables, respectively. The results showed that in the first investment round (*F*(1, 148) = 4.46, *p* = .036, *η* ^2^ = 0.03), the group main effect was significant, the investment amount in the experimental group was significantly higher than that in the control group (*M*_R1 of experimental_−*M*_R1 of control_ = 10.71), and the interaction between gender and group was significant (*F*(1, 148) = 4.24, *p* = .041, *η* ^2^ = 0.03). There was no significant difference in the amount of first‐round investment for females in the experimental and control groups; however, there was a significantly higher first‐round investment amount for males in the experimental group than in the control group (*M*_experimental R1 of males_−*M*_control R1 of females_ = 21.15). The group main effect (*F*(1, 148) = 1.28, *p* = .259, *η* ^2^ = 0.00) and interaction effect (*F*(1, 148) = 0.54, *p* = .463, *η* ^2^ = 0.00) were not found for the average of the six rounds of investment. The results revealed that empathy elicitation significantly enhanced the participants' cooperative behavior, and that males were more likely to be influenced by the empathic state to make their cooperative decision‐making.

### Discussion

Previous studies have shown that a higher level of empathy is strongly associated with social competence and prosocial behavior throughout life. However, the previous research on empathy and cooperation has yielded inconsistent results, and the role of gender in this relationship remains unclear. This study focused on junior high school adolescents. Study 1 explored the relationship between the adolescents' empathy and cooperative propensity, as well as the gender differences, from the empathic trait perspective. Study 2 further used a short‐term longitudinal design to verify this relationship and identify gender differences based on the results of Study 1. Study 3 explored the effect of empathy on cooperative behavior from the empathic state perspective and examined the role of gender. This study found that adolescents' empathy had a facilitative effect on their cooperative propensity and cooperative behavior, and that there were gender differences in this facilitative effect, with empathy being more predictive of cooperation for males than for females. The findings hold significant implications for generalizing the empathy–altruism hypothesis and recognizing gender differences in the influence of empathy on social interactions, as well as for implementing cooperative education among adolescents from the empathic perspective.

#### Relationship between empathy and cooperation

Most researchers have generally agreed that empathy is an important motivator for prosocial behavior, and that beneficial perspective taking and emotional responsiveness enable individuals to better understand others' needs (Yang et al., 2014). Empirical studies have found that at the between‐person level, people with higher levels of empathy tend to have more prosocial behaviors (de Waal, 2008), are more attentive to others' emotional changes (Yan et al., 2016), and have better social adaptiveness. At the within‐person level, adolescents who report higher empathy levels than usual describe having more prosocial behaviors than usual 1 year later (Carrizales et al., 2021).

The current study's results were consistent with the expectations, indicating a positive relationship between empathy and cooperation among adolescents. Specifically, empathy facilitated cooperation. This supports the empathy–altruism hypothesis in terms of empathy's facilitation of cooperation, which is a subtype of prosocial behavior (Batson et al., 1991), and has implications for the generalization of the empathy–altruism hypothesis.

Furthermore, this study combined cooperative propensity and behavior to validate the integrative model of decision‐making (Parks et al., 2013) and the dynamic model theory of empathy (Liu et al., 2009). From the trait perspective, individuals who are adequate at empathizing tend to have higher levels of social mindfulness, which serves as the foundation for building trust and fostering cooperative intention (Dou et al., 2018). From the situational perspective, empathy serves as an important communication facilitator, allowing individuals to perceive the distress of others and construct common feelings and perceptions between themselves and others. This encourages adolescents to focus on others, enabling them to subsequently make decisions and engage in behaviors that are beneficial for others or the collective during interpersonal interactions.

However, in this study, empathy induction only increased the adolescents' cooperative behavior indicator in the first investment round in the public goods dilemma game, while the average amount of the six rounds of investment did not significantly differ between the groups. A possible reason is that although six rounds of the public goods dilemma game were conducted in this study, the interactive objects of each round were randomly matched. That is, participants faced different interactive objects in each round of investment, and they were unable to accurately identify the intentions of their cooperative objects (Cui et al., 2022). Similarly, previous studies found that the level of individual cooperation tends to decrease when information about the interaction target is unclear, even if the interaction is repeated (Van Lange et al., 2013). In the current study, however, the participants in the game were given timely feedback on the behavioral benefits of the previous round's decisions, which they could have used as the basis for the subsequent round's decisions. Therefore, induced empathy is not only time‐sensitive but is also disturbed by the feedback of the first‐round cooperation benefits and the uncertainty of the interaction objects. Thus, the first round of investment in this study may have better reflected the participants' cooperation as being induced by empathy. Further research is required to explore how adolescents after empathy induction interact cooperatively with others using a fixed cooperative object approach.

#### Gender differences in the relationship between empathy and cooperation

Study 1 used a questionnaire survey to reveal that male adolescents exhibited a stronger correlation between empathy levels and cooperative propensities than did females. In Study 2, short‐term longitudinal data from eighth‐grade adolescents further corroborated this finding. Study 3 confirmed that empathy‐induced emotional responses were more likely to enhance cooperative behavior in the public goods dilemma among males than among females. In sum, the relationship between empathy and cooperation among junior high school adolescents varied with gender, and empathy was a better predictor of cooperation among male eighth graders.

The gender differences among the eighth graders could be attributed to the following factors. During puberty, adolescents experience rapid physical and psychological development, as well as significant changes in social roles, which coincide with the differentiation of physiological and psychological characteristics between males and females (Hyde, 2014). Previous research has indicated that gender differences vary depending on the environment and become more pronounced with age between childhood and adolescence (Chaplin & Aldao, 2013). Moreover, gender differences in empathy are influenced by age, with the gender gap peaking during adolescence (Yan & Su, 2018). Eighth‐grade students are in the mid‐stage of adolescence (Qiu et al., 2021) and have already experienced rapid development, so individual and gender differences will become more apparent and exhibit relatively greater variability.

There may also be several reasons for the finding that empathy among male adolescents is more predictive of cooperation than that among female adolescents. On the one hand, females have higher initial levels of empathy that, after reaching school age, develop faster and reach even higher levels (Chen et al., 2014). However, their cooperative propensity is relatively highly stable and may not develop as fast as empathy, so the longitudinal predictive relationship between the two aspects is broken.

On the other hand, we attempted to conduct a cross‐lagged analysis on the longitudinal data of Study 2 to provide possible explanations for the predictive relationship between empathy and cooperation across genders, and we found that the predictive pathway between empathy and cooperative propensity exhibited opposite directions for males and females. Specifically, for males, stronger empathy was associated with greater cooperative propensity, whereas the opposite result was found for females. This exploratory analysis further contributes to an understanding of the observed stronger predictive effect of empathy on cooperation among males than among females found herein. The relationship theory suggests that females have a stronger desire to develop and maintain personal relationships with others than do males (Portman et al., 2011), value interpersonal relationships more, and focus on already established social relationships in social interactions; moreover, members in intimate female relationships are often encouraged to share their respective emotional experiences or feelings. Thus, females may be more likely to develop behavioral aspects from interpersonal relationships as a starting point, as shown in the results of the cross‐lagged analysis, which reveals the need for cooperation before empathy. This is consistent with Van Der Graaff et al.'s (2018) finding that the predictive effect of prosocial behavior on empathic concern is established only in females.

#### Limitations and future directions

This study validated the facilitating effect of empathy on cooperation among Chinese junior high school adolescents, and examined the role of gender in this relationship.

However, this study had several limitations. First, this study primarily investigated the relationship between empathy and cooperative propensity among adolescents, as well as the gender differences, from the trait and state perspectives. It did not, however, extensively delve into the specific relationships between different components of empathy (e.g., affective and cognitive components) and cooperation. As a response to this limitation, this study conducted exploratory analyses using data from Studies 1 and 2 by subdividing the empathy components and cooperation dimensions. These results are included in the Supplementary Materials but are not fully explored or discussed herein. Expanding research to examine the specific dimensions and components of empathy in relation to cooperation could be an intriguing and complex independent study (Kim et al., 2020). Moreover, the underlying psychological and physiological mechanisms through which empathy influences cooperation also warrant further investigation. Future studies could use questionnaires, behavioral experiments, and functional magnetic resonance imaging or near‐infrared imaging techniques to reveal the in‐depth impact of empathy on cooperation.

Second, the sample size in Study 2 was relatively small, and the study only selected a portion of eighth‐grade students from one junior high school as participants. This may limit the generalizability of the findings. Furthermore, this study had a relatively short follow‐up period of only 4 months, allowing for the examination of only short‐term effects of empathy on cooperative propensity. Future research could optimize the study's design by increasing the follow‐up period, extending the follow‐up interval, and expanding the sample size for a more comprehensive examination of the stable and long‐term effects and influence patterns.

Finally, this study only used the public goods dilemma paradigm to explore cooperative behavior, and each interaction round was randomly matched with group members, which was, to some extent, repetitive and did not reflect the interaction pattern between participants. It also may have interfered with the effect of empathy elicitation on behavior. Future research could select diverse paradigms of cooperative behavior or compare repetitive and non‐repetitive approaches to optimize the exploration of cooperative behavior.

## CONFLICT OF INTEREST STATEMENT

The authors declare that there are no conflicts of interest.

## ETHICS STATEMENT

All study participants provided written informed consent. The study design was approved by the Ethics Review Board of the Social Sciences Office of Shanghai Normal University.

## Supporting information


**Data S1.** Supporting information.

## Data Availability

Data are available from the corresponding author upon reasonable request.
