# Supplementary material for: Empathy and cooperation vary with gender in Chinese junior high school adolescents
Source: Psych J. 2023 Nov 9;13(1):55–65. doi: 10.1002/pchj.705 (PMC10917101; doi:10.1002/pchj.705)
Supplement: Supplementary file 1 — Data S1. Supporting information. [file PCHJ-13-55-s001.docx]

**Supplementary Materials** **for**

**Empathy and cooperation vary with gender in Chinese junior high school adolescents**

In Study 1 and Study 2, this study divided empathy into cognitive and emotional components, and examined the relationship between these different empathy components and various cooperative propensity dimensions, as well as the gender differences, among eighth-grade adolescents.

**Study 1**

The descriptive statistical analysis results of Study 1 are shown in Table 1.

**Table 1**

*Descriptive statistics and correlation analysis of each dimension*

|  | *M* | *SD* | 1 | 2 | 3 | 4 | 5 |
| --- | --- | --- | --- | --- | --- | --- | --- |
| 1. Gender | 1.55 | .50 | - |  |  |  |  |
| 2. Cognitive empathy | 3.29 | .70 | .19^**^ | - |  |  |  |
| 3. Emotional empathy | 3.19 | .59 | .07 | .14^**^ | - |  |  |
| 4. Inclusiveness | 4.02 | .68 | .05 | .31^***^ | .14^**^ | - |  |
| 5. Reciprocity | 3.14 | .79 | -.11 ^*^ | .13^**^ | .21^***^ | .29^***^ | - |
| 6. Gregariousness | 3.75 | .78 | -.09 | .09 | .21^***^ | .37^***^ | .62^***^ |

*Note*. *N* = 448; gender (0 = male, 1 = female); ^*^*p* < .05, ^**^*p* < .01, ^***^*p* < .001.

The analysis results of the association between cognitive and emotional empathy and cooperative propensity, as well as the role of gender, are shown in Table 2.

**Table 2**

*The association between cognitive and emotional empathy on the various cooperative propensity dimensions of junior high school adolescents, and the moderating role of gender*

| Variable | | Effect | *SE* | *t* | 95%CI | |
| --- | --- | --- | --- | --- | --- | --- |
|  |  |  |  |  | LLCI | ULCI |
| Inclusiveness | |  |  |  |  |  |
|  | Constant | .00 | .07 | -0.04 | -.14 | .13 |
|  | Cognitive empathy | .36 | .07 | 5.60^***^ | .24 | .49 |
|  | Gender | -.05 | .09 | -0.55 | -.23 | .13 |
|  | Cognitive empathy*Gender | -.11 | .09 | -1.25 | -.29 | .06 |
| Reciprocity | |  |  |  |  |  |
|  | Constant | .10 | .07 | 1.34 | -.05 | .24 |
|  | Cognitive empathy | .23 | .07 | 3.36^**^ | .09 | .36 |
|  | Gender | -.30 | .09 | -3.25^**^ | -.49 | -.12 |
|  | Cognitive empathy*Gender | -.14 | .09 | -1.51 | -.32 | .04 |
| Gregariousness | |  |  |  |  |  |
|  | Constant | .06 | .07 | 0.85 | -.08 | .21 |
|  | Cognitive empathy | .24 | .07 | 3.47^***^ | .10 | .37 |
|  | Gender | -.28 | .10 | -2.95^**^ | -.47 | -.09 |
|  | Cognitive empathy*Gender | -.24 | .09 | -2.58^*^ | -.43 | -.06 |
| Inclusiveness | |  |  |  |  |  |
|  | Constant | -.15 | .07 | -2.29^*^ | -.28 | -.02 |
|  | Emotional empathy | .29 | .07 | 4.36^***^ | .16 | .42 |
|  | Gender | .08 | .09 | 0.91 | -.09 | .25 |
|  | Emotional empathy*Gender | -.29 | .09 | -3.27^**^ | -.46 | -.12 |
| Reciprocity | |  |  |  |  |  |
|  | Constant | .01 | .07 | 0.10 | -.12 | .13 |
|  | Emotional empathy | .21 | .07 | 3.17^**^ | .08 | .34 |
|  | Gender | -.24 | .09 | -2.74^**^ | -.41 | -.07 |
|  | Emotional empathy*Gender | .00 | .09 | -0.03 | -.18 | .17 |
| Gregariousness | |  |  |  |  |  |
|  | Constant | -.03 | .07 | -0.53 | -.17 | .10 |
|  | Emotional empathy | .18 | .07 | 2.59^**^ | .04 | .31 |
|  | Gender | -.21 | .09 | -2.30^*^ | -.38 | -.03 |
|  | Emotional empathy*Gender | .05 | .09 | 0.55 | -.13 | .23 |

*Not*e. CI = confidence interval, LLCI = lower limit of confidence interval, ULCI = upper limit of confidence interval; *N* = 448; gender (0 = male, 1 = female); ^*^*p* < .05, ^**^*p* < .01, ^***^*p* < .001.

**Study 2**

The descriptive statistical analysis results of Study 2 are shown in Table 3.

**Table 3**

*Descriptive statistics and correlation analysis of each dimension* *at T1 and T2*

|  | *M* | *SD* | 1 | 2 | 3 | 4 | 5 | 6 | 7 | 8 | 9 | 10 |
| --- | --- | --- | --- | --- | --- | --- | --- | --- | --- | --- | --- | --- |
| 1. Gender | 0.50 | .50 | - |  |  |  |  |  |  |  |  |  |
| 2. T1 cognitive empathy | 3.03 | .65 | .03 | - |  |  |  |  |  |  |  |  |
| 3. T1 emotional empathy | 3.22 | .56 | .08 | -.01 | - |  |  |  |  |  |  |  |
| 4. T1 inclusiveness | 4.05 | .64 | -.01 | .30^***^ | .17^*^ | - |  |  |  |  |  |  |
| 5. T1 reciprocity | 3.14 | .71 | -.07 | -.02 | .22^**^ | .20^**^ | - |  |  |  |  |  |
| 6. T1 gregariousness | 3.87 | .64 | -.04 | .11 | .17^*^ | .35^***^ | .56^***^ | - |  |  |  |  |
| 7. T2 cognitive empathy | 3.57 | .56 | .05 | .26^***^ | .20^**^ | .29^***^ | .15^*^ | .17^*^ | - |  |  |  |
| 8. T2 emotional empathy | 3.17 | .56 | .08 | -.01 | .33^***^ | .04 | .13 | .13 | .24^***^ | - |  |  |
| 9. T2 inclusiveness | 3.85 | .89 | .02 | .19^**^ | .15^*^ | .43^***^ | .13 | .12 | .40^***^ | .08 | - |  |
| 1. T2 reciprocity | 3.87 | .74 | .01 | .12 | .18^**^ | .31^***^ | .41^***^ | .31^***^ | .15^*^ | .31^***^ | .32^***^ | - |
| 11. T2 gregariousness | 3.66 | .72 | -.04 | .16^*^ | .19^**^ | .29^***^ | .28^***^ | .47^***^ | .22^**^ | .17^*^ | .41^***^ | .59^***^ |

*Note*. *N* = 218; gender (0 = male, 1 = female); ^*^*p* < .05, ^**^*p* < .01, ^***^*p* < .001.

Table 4 presents the analysis results of the predictive relationship between cognitive and emotional empathy and the cooperative propensity dimensions, and the moderating role of gender, in the longitudinal data of Study 2.

**Table 4**

*The prediction of cognitive and emotional empathy at T1 on the various cooperative propensity dimensions at T2 among junior high school adolescents, and the moderating role of gender*

| Variable | | Effect | *SE* | *t* | 95%CI | |
| --- | --- | --- | --- | --- | --- | --- |
|  |  |  |  |  | LLCI | ULCI |
| T2 inclusiveness | |  |  |  |  |  |
|  | Constant | -.02 | .09 | -0.19 | -.19 | .16 |
|  | T1 inclusiveness | .40 | .07 | 6.18^***^ | .27 | .53 |
|  | Cognitive empathy | .08 | .09 | 0.95 | -.09 | .26 |
|  | Gender | .03 | .12 | 0.28 | -.21 | .28 |
|  | Cognitive empathy*Gender | -.04 | .12 | -0.31 | -.28 | .21 |
| T2 reciprocity | |  |  |  |  |  |
|  | Constant | -.03 | .09 | -0.30 | -.20 | .15 |
|  | T1 reciprocity | .41 | .06 | 6.56^***^ | .29 | .53 |
|  | Cognitive empathy | .18 | .08 | 2.07^*^ | .01 | .34 |
|  | Gender | .06 | .12 | 0.45 | -.19 | .30 |
|  | Cognitive empathy*Gender | -.10 | .12 | -0.84 | -.35 | .14 |
| T2 gregariousness | |  |  |  |  |  |
|  | Constant | .02 | .08 | 0.29 | -.14 | .19 |
|  | T1 gregariousness | .44 | .06 | 7.39^***^ | .33 | .56 |
|  | Cognitive empathy | .22 | .08 | 2.68^**^ | .06 | .38 |
|  | Gender | -.04 | .12 | -0.35 | -.28 | .19 |
|  | Cognitive empathy*Gender | -.24 | .12 | -1.98^*^ | -.47 | .00 |
| T2 inclusiveness | |  |  |  |  |  |
|  | Constant | .00 | .09 | -0.02 | -.17 | .17 |
|  | T1 inclusiveness | .41 | .06 | 6.54^***^ | .28 | .53 |
|  | Emotional empathy | .21 | .08 | 2.52^*^ | .05 | .38 |
|  | Gender | .03 | .12 | 0.22 | -.21 | .27 |
|  | Emotional empathy*Gender | -.30 | .12 | -2.41^*^ | -.54 | -.05 |
| T2 reciprocity | |  |  |  |  |  |
|  | Constant | -.02 | .09 | -0.20 | -.19 | .16 |
|  | T1 reciprocity | .39 | .06 | 6.03^***^ | .26 | .51 |
|  | Emotional empathy | .15 | .09 | 1.74 | -.02 | .32 |
|  | Gender | .04 | .12 | 0.35 | -.20 | .29 |
|  | Emotional empathy*Gender | -.11 | .13 | -0.90 | -.36 | .13 |
| T2 gregariousness | |  |  |  |  |  |
|  | Constant | .04 | .08 | 0.42 | -.13 | .20 |
|  | T1 gregariousness | .45 | .06 | 7.48^***^ | .33 | .57 |
|  | Emotional empathy | .21 | .08 | 2.59^*^ | .05 | .38 |
|  | Gender | -.05 | .12 | -0.44 | -.29 | .18 |
|  | Emotional empathy*Gender | -.22 | .12 | -1.85 | -.46 | .02 |

*Not*e. CI = confidence interval, LLCI = lower limit of confidence interval, ULCI = upper limit of confidence interval; *N* = 218, gender (0 = male, 1 = female); ^*^*p* < .05, ^**^*p* < .01, ^***^*p* < .001.

The results of Study 1 and Study 2 revealed gender differences in the relationship between cognitive empathy and gregariousness, as well as between affective empathy and inclusiveness. Specifically, for the male adolescents, cognitive empathy had a significant predictive effect on gregariousness (Study 1: *β* = .24, *p* < .001; Study 2: *β* = .22, *p* = .008), and affective empathy significantly predicted inclusiveness (Study 1: *β* = .29, *p* < .001; Study 2: *β* = .21, *p* = .013). However, no significant relationships were observed among the female adolescents.
